# PROtocolized Care to Reduce HYpotension after Spinal Anesthesia (ProCRHYSA randomized trial): statistical plan

**DOI:** 10.1186/2036-7902-7-S1-A3

**Published:** 2015-03-09

**Authors:** Samuele Ceruti, Sergio De Vivo, Mattia Peruzzo, Denis De Bianchi, Luciano Anselmi, Andrea Saporito

**Affiliations:** 1 Department of Intensive Care Unit, Ospedale Regionale di Bellinzona, Via Ospedale 12, 6500 Bellinzona, Switzerland; 2 Department of Anesthesiology, Ospedale Regionale di Bellinzona – Via Ospedale 12, 6500 Bellinzona, Switzerland

## Background

Spinal anesthesia is a regional anesthesia technique widely employed in clinical practice. The common side effects consist in a reduction of systemic vascular resistances, with systemic hypotension. To prevent this complication, blind administration of fluids is commonly used. This is accomplished on an empirical basis, carrying risk of possible volume overload. Vena cava ultrasound has been shown to be an effective method to assess fluid responsiveness in critical care patients, however this method has never been studied in a non critical population. Aim of this study is to assess the efficacy of vena cava ultrasound guided titrated volume repletion in preventing spinal anesthesia induced hypotension in an elective surgical population.

## Objective

To state our analysis plan for trial data.

## Methods

We designed a randomized, case-control, prospective trial comparing standard practice to ultrasound guided vena cava fluid repletion before spinal anesthesia for elective surgery. After written informed consent, we randomized ASA 1 to 3 patients into two groups: the first received no preliminary volume repletion, according to standard local practice, while the second was assessed with vena cava echography before spinal anesthesia. Patients found to be responsive to fluids were given subsequent boluses of 500 ml crystalloids before proceeding to spinal anesthesia and reassessed afterward until found euvolemic. Non invasive arterial pressure was periodically measured in any patient after spinal anesthesia until discharge to recovery room and the significant hypotension rate was subsequently calculated in the two groups.

Significant hypotension was defined according to international guidelines as fall in systolic arterial blood pressure more than 50 mmHg or 25% from baseline value, an absolute value of systolic pressure less than 80 mmHg, an absolute value of mean pressure less than 60 mmHg, a reduction in mean arterial pressure more than 30% from baseline value and/or clinical symptoms of inadequate perfusion[i]. Exclusion criteria were considered contraindication to spinal anesthesia, patientâ€™s refusal or lack of protocol adherence. Data are given as percentage of significant hypotension, p value (p) and confidence intervals (CI). Power calculation was preliminary performed, identifying a sample size of 150 patients per group (95% confidence level) .

## Results

A total of 64 patients were recruited in a 5 months period. 16 patients did not meet inclusion criteria and were excluded. 19 patients randomized to the cases group were subsequently excluded because of protocol adherence issues (poor echographic windows did not consent adequate vena cava diameter measurement). Post-spinal significant hypotension rate was 66% in control group (N=32) and 45% in the group whose preventive volume repletion was guided through vena cava ultrasound (N=32), this difference being statistically significant (p=0,043 CI=95%).

## Conclusions

Vena cava ultrasound assessment is an effective method to assess fluid responsiveness also in non critical patient who underwent preoperative fasting before elective surgery and can be routinely employed for guiding titrated and tailored preoperative volume repletion in order to reduce risk of significant hypotension after spinal anesthesia.

## Trial registration

The trial is registered on http://www.clinicaltrials.gov with number NCT02070276.
